# COVEPIC (Cognitive and spOrt Virtual EPIC training) investigating the effects of home-based physical exercise and cognitive training on cognitive and physical functions in community-dwelling older adults: study protocol of a randomized single-blinded clinical trial

**DOI:** 10.1186/s13063-021-05476-2

**Published:** 2021-07-29

**Authors:** Emma Gabrielle Dupuy, Florent Besnier, Christine Gagnon, Thomas Vincent, Catherine-Alexandra Grégoire, Caroll-Ann Blanchette, Kathia Saillant, Nadia Bouabdallaoui, Josep Iglesies-Grau, Marie Payer, Marie-France Marin, Sylvie Belleville, Martin Juneau, Paolo Vitali, Mathieu Gayda, Anil Nigam, Louis Bherer

**Affiliations:** 1 Research center and Centre ÉPIC, Montreal Heart Institute, Université de Montréal, Montréal, Québec H1T 1 N6 Canada; 2 Department of Medicine, Faculty of Medicine, Université de Montréal, Montréal, Québec H3C 3 J7 Canada; 3 Department of Psychology, Université du Québec à Montréal, Montréal, Québec H3C 3P8 Canada; 4 Research Center, Institut universitaire en santé mentale de Montréal, Montréal, Québec H1N 3 M5 Canada; 5 Research Center, Institut Universitaire de Gériatrie de Montréal, Montréal, Québec H3W 1 W5 Canada; 6 Department of Psychology, Université de Montréal, Montréal, Québec H2V 2S9 Canada; 7 CIUSSS Nord-de-l’Île-de-Montréal, Montréal, Québec Canada; 8 Department of Neurology and Neurosurgery, Faculty of Medicine, McGill University, Montréal, Québec Canada

**Keywords:** Aging, Cognition, Cognitive training, Physical exercise, Remote monitoring, Home-based training, COVID

## Abstract

**Background:**

In the context of the COVID-19 pandemic, lockdown and social distancing measures are applied to prevent the spread of the virus. It is well known that confinement and social isolation can have a negative impact on physical and mental health, including cognition. Physical activity and cognitive training can help enhance older adults’ cognitive and physical health and prevent the negative collateral impacts of social isolation and physical inactivity. The COVEPIC study aims to document the effects of 6 months of home-based physical exercise alone versus home-based physical exercise combined with cognitive training on cognitive and physical functions in adults 50 years and older.

**Methods:**

One hundred twenty-two healthy older adults (> 50 years old) will be recruited from the community and randomized to one of the two arms for 6 months: (1) home-based physical exercises monitoring alone and (2) combined physical exercises monitoring with home-based cognitive training. The primary outcome is cognition, including general functioning (Montreal Cognitive Assessment (MoCA) score), as well as executive functions, processing speed, and episodic memory (composite *Z*-scores based on validated neuropsychological tests and computerized tasks). The secondary outcome is physical functions, including balance (one-leg stance test), gait and mobility performance (Timed Up and Go, 4-meter walk test), leg muscle strength (5-time sit-to-stand), and estimated cardiorespiratory fitness (Matthews’ questionnaire). Exploratory outcomes include mood, anxiety, and health-related quality of life as assessed by self-reported questionnaires (i.e., Geriatric depression scale-30 items, Perceived stress scale, State-trait anxiety inventory-36 items, Perseverative thinking questionnaire, Connor-Davidson Resilience Scale 10, and 12-item Short Form Survey).

**Discussion:**

This trial will document the remote monitoring of home-based physical exercise alone and home-based physical combined with cognitive training to enhance cognitive and physical health of older adults during the COVID-19 pandemic period. Remote interventions represent a promising strategy to help maintain or enhance health and cognition in seniors, and potentially an opportunity to reach older adults in remote areas, where access to such interventions is limited.

**Trial registration:**

Clinical trial Identifier NCT04635462. COVEPIC was retrospectively registered on November 19, 2020.

## Background

The current COVID-19 pandemic resulted in unusual public health measures challenging the worldwide population's lifestyle. To limit the spread of the virus, confinement and social distancing measures are the cornerstone of most public health policies all around the world and are strongly recommended by the World Health Organization (WHO). Older adults represent a group at higher risk of hospitalization and death from COVID-19 [1–3], for whom social distancing measures are of paramount importance. However, refraining from outings for a long period implies an important reduction in social interactions and a substantial increase in physical inactivity and sedentary behaviors likely to impact the populations’ health. The negative psychological impacts of quarantine and lockdown have been recently documented [4, 5], and social isolation and loneliness (i.e., perceived social isolation) are known to be risk factors for anxiety, depression, and cognitive decline [6]. In older adults, social isolation and loneliness have also been associated with poorer cognitive functions [7]. Additionally, sedentary behaviors and physical inactivity are associated with an increased risk of cardiovascular events, rehospitalization, and decreased cardiorespiratory fitness [8, 9]. Consequently, the important changes in daily-life routine induced by the COVID-19 crisis may impact older adults’ health as they are more likely to be suffering from chronic conditions [10]. Therefore, adapted and effective solutions are needed to enhance physical health and cognition in seniors while maintaining social distancing during this pandemic period.

The Global Recommendations of the WHO, recently updated, state the importance of physical activity to promote health outcomes across the lifespan, highlight the fact that short bouts of physical activity are better than none, and provide new guidelines to reduce sedentary behavior [11]. Moreover, to fight against the mental and physical consequences of the COVID-19 pandemic, governmental health authorities, as well as the scientific community, recommend to stay physically active [8]. Multiple studies and meta-analyses suggest that physical activity and exercise have a positive impact on physical condition [12–14] and cognition [15–17], especially on executive functions [18, 19]. Recent evidence demonstrated that performing physical activity, even at low intensity, could help reduce some of the negative consequences of social distancing on older adults’ mental health [20]. In the current pandemic context, web-based and remote interventions are a promising solution, as they have previously demonstrated robust effectiveness in promoting physical activity [21]. In their systematic review, Gaeredts et al. [22] highlight the potential of remote training with a coach to promote physical activity and to enhance older adults' physical capacity. However, a limited number of studies have investigated the impact of long-term remote monitoring of physical exercise on physical and mental health, and the potential benefits on cognition have not been investigated. Moreover, the optimal dose of home-based physical exercise to promote older adults’ cognitive and physical health remains to be investigated, especially in a pandemic context likely to increase sedentary behaviors and social isolation.

Computerized cognitive training has also been used to enhance specific aspects of cognitive functioning in seniors [23]. Moreover, cognitive training seems to improve postural control [24] and mobility [25], suggesting that it can help enhance and maintain gross motor skills, including balance and mobility-related outcomes [25]. Physical activity and cognitive training are thought to involve different mechanisms of brain plasticity. Recent studies investigating the combined effect of physical exercise and cognitive training suggest that they exert synergistic effects on cognition [26, 27]. Hence, a combined intervention involving both home-based cognitive and physical training could lead to an even more pronounced improvement of cognitive health and mobility than physical exercise alone in older adults.

This study will investigate the benefits of monitoring home-based physical exercise combined with cognitive training in community-dwelling older adults and will explore the dose-response relationship (i.e., total amount of exercise including type, frequency, duration, and intensity) of home-based physical exercise for optimal cognitive and physical health benefits. More precisely, this trial aims to (1) document the effects of remote monitoring of home-based physical exercise on cognitive and physical functions in older adults, and (2) address the added benefits of a multidomain home-based intervention involving both cognitive training and physical exercise.

### Hypotheses

1. After 6 months of home-based physical exercise, participants will show an improvement in cognitive (primary outcome) and physical functions measures (secondary outcomes), with larger gains in executive functions.
2. Participants in the combined home-based physical exercise and cognitive training group will show greater improvements in cognition and physical functions post-intervention compared to participants who did not complete the cognitive training.

## Methods/design

### Design

COVEPIC is a randomized, single-blind trial with two parallel intervention arms:

- *Arm 1 – Physical exercise*: remote monitoring and counseling of home-based physical exercise.
- *Arm 2 – Physical exercise and cognitive training*: remote monitoring and counseling of home-based physical exercise and home-based cognitive training.

**Fig. 1:**
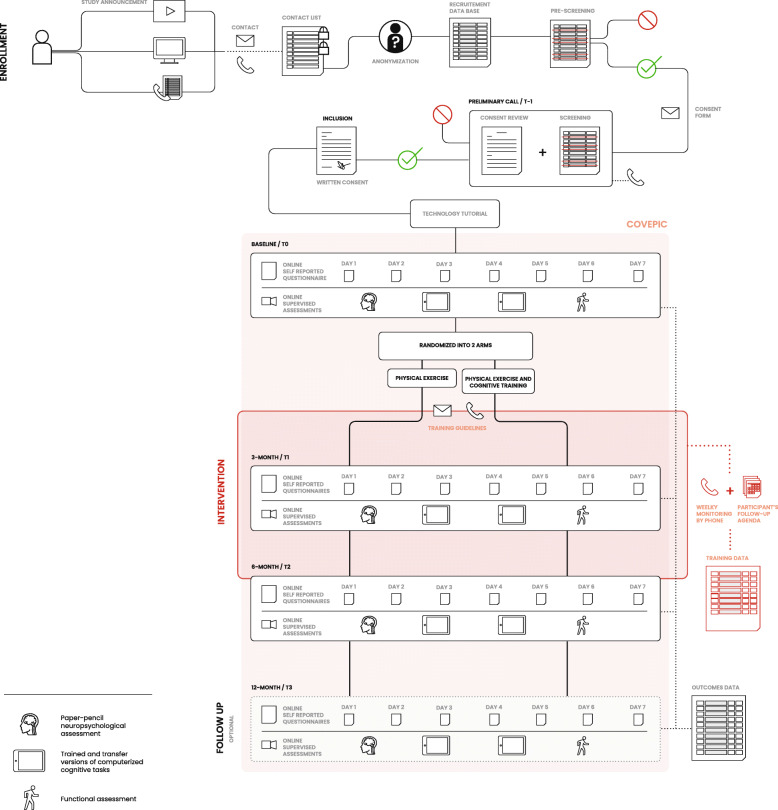
Study design, remote setting and procedures of the trial, and online data management of the trial

The study design includes six months of intervention. All participants will be assessed at baseline, 3 months, and 6 months. At each timepoint, four testing sessions will be done remotely using videoconference supervision and online questionnaires over a period of 7–10 days. At twelve months (6 months after the end of the intervention), a follow-up assessment will be performed to explore the potential maintenance of intervention effects. Participation in this follow-up assessment will be left to the participant's discretion, as it does not directly address the main hypotheses. The trial was designed according to SPIRIT guidelines (cf. SPIRIT checklist in the additional file) [28]. Figure 1 illustrates the trial design according to items of SPIRIT figure, which is also available in additional files.

### Participants

A total of 122 participants aged 50 and older will be enrolled according to the following inclusion criteria:

- Have access to a tablet (i.e., iPad or Android) or a computer with an Internet connection,
- Absence of significant cognitive impairment (i.e., score of 19/23 or higher on the telephone version of Mini-Mental State Examination, MMSE) [29],
- Absence of non-cardiopulmonary limitation to exercise (e.g., arthritis) or of a severe exercise intolerance,
- Absence of respiratory disease (e.g., severe asthma, COPD, severe COVID-19 related symptoms) as documented with a medical questionnaire,
- Absence of cardiovascular disease (e.g., chronic heart failure, somatic aortic stenosis, atrial fibrillation, malignant arrhythmias, any documented atherosclerosis disease) as documented with a medical questionnaire.

No systematic dropout criteria are fixed as dose-response relationships will be explored.

### Remote setting and procedures, online data management

There is no on-site visit planned in the course of the study. The research team has implemented online tools that allow performing all assessments, exercise monitoring, and cognitive training entirely remotely. The research project will be advertised to active members of physical exercise centers and to community-dwelling adults through online study announcements (e.g., videos, interviews, newsletters). Potential participants will be able to express their interest by leaving a voice mail or sending an email to an email address dedicated to the study. The name and contact information of potential participants will be compiled in a password-protected encrypted file located in a cloud folder with restricted access. The anonymization table is built at this stage, where each contact is associated with an anonymous study I.D. number. A two-stage screening process will be used to select participants (timepoint T-1; Table 1). Initial contact will be made over the phone by a member of the research team. During this pre-screening call, only potential participants with no major physical or medical limitations to exercise and with access to the Internet and necessary technology will be selected. They will then receive the information and consent form by email, and the preliminary call will be scheduled with a member of the research team. During the preliminary call, the information and consent form will be presented to the potential participants, and they will be given the opportunity to provide oral consent. They will then be required to send written consent by email. If oral consent is obtained, the cognitive (MMSE) and physical (PARQ+) screenings will then be performed with a research assistant and will later be reviewed by a neuropsychologist and a kinesiologist, respectively. Once the research team receives the written consent by email and ensures eligibility, the participant will be considered enrolled. A technology tutorial will then be scheduled to ensure that Internet access and tools are ready and sufficiently mastered by participants. Collection of measurements and training data all along the study will be managed by assessors and trainers using online case report forms specifically dedicated to each testing session. Participants will receive automatic standardized communications by email to transmit study information like appointment schedules, links to self-reported questionnaires, and training guidelines. A non-overwriting process will ensure data integrity: all form submissions will be recorded in a journal fashion. Moreover, all data entries will be traceable and associated with a timestamp along with the identity of the research team member who completed the data entry. To ensure the blinding of the study, data collection will be divided into two sections on the online platform: training and evaluations. Research team members will receive different access based on their roles. The above-described procedure is summarized in Fig. 1.

**Table 1:** List of assessments across the study timepoints according to the remote method used

Remote method	Domain	Procedure	T-1	T0	T1	T2	T3
**Phone call**	**-**	Written informed consent	X				
		Mini-Mental State Examination	X				
		Physical activity readiness questionnaire	X				
		Technology tutorial	X				
**Online videoconference**	**Cognition**	Montreal Cognitive Assessment		X	X	X	X
		Oral Trail Making Test		X	X	X	X
		Verbal Fluency Test		X	X	X	X
		Digit Span test		X	X	X	X
		Similarity Test		X	X	X	X
		Rey auditory verbal learning test		X	X	X	X
		Computerized trained cognitive task		X	X	X	X
		Computerized transfer cognitive task		X	X	X	X
	**Physical functions**	One leg balance test		X	X	X	X
		5-time Sit-to-stand test		X	X	X	X
		Timed Up and Go test		X	X	X	X
		4-meter walking speed test		X	X	X	X
**Online self-reported forms**		Matthews Questionnaire		X	X	X	X
	**Quality of life**	12-Item Short Form Survey		X	X	X	X
	**Mood and anxiety**	State Anxiety Inventory		X	X	X	X
		Geriatric Depression Scale		X	X	X	X
		Perceived Stress Scale		X	X	X	X
		Perseverative Thinking Questionnaire		X	X	X	X
		Connor-Davidson Resilience Scale 10		X	X	X	X
		Intolerance of Uncertainty Scale		X			
		Trait Anxiety Inventory		X			
		Anxiety sensitivity Index		X			
	**Social environment**	Social and Community Involvement Questionnaire		X	X	X	X
		Lubben Social Network Scale		X	X	X	X
	**Sleep**	Pittsburgh Sleep Quality Index		X	X	X	X
		Berlin Questionnaire		X	X	X	X
	**Nutrition**	Short Diet Questionnaire		X			X
	**Prior Physical activity**	Physical Activity Scale for the Elderly		X			
	**Cognitive reserve**	Cognitive reserve Questionnaire		X			
	**Gender**	Bem Sex-Role Inventory		X			
	**Medical history**	Medical Questionnaire		X			
	**COVID-19 repercussions**	QCOVID Questionnaire		X			

### Interventions

After being randomly (see the “Randomization and blinding” section) assigned to one of the 2 study arms, participants will receive a group-specific training guide. The training guide contains recommendations about the nature, intensity, and frequency of the training sessions. Participants will then attend an introductory session over the phone given by their training coach. Participants will be invited to follow the training guide while adjusting their dose as they see fit.

#### Physical exercise

Participants will be encouraged to complete a home-based physical exercise using video capsules available via Facebook or Youtube, created by the Centre ÉPIC of the Montreal Heart Institute’s team of kinesiologists. The videos last approximately 15 min and include a 3- to 5-min warm-up, followed by a 10-min training, and finally by a 2-min cool-down period (https://tinyurl.com/epic-icm). The exercises do not require any equipment and comprise aerobic, muscular strengthening, flexibility, and/or balance exercises. For each exercise, several difficulty levels are described to allow participants to perform the exercise according to their physical capacities. Participants will be invited to perform exercise sessions at least 5 times per week and could be encouraged to do more during the weekly monitoring. This recommendation aims to provide a target for a minimal amount of physical exercise [30] that all participants can introduce and autonomously maintain in their routine regardless of the sanitary context, their initial physical condition, or prior exercise routine. The exercise sessions can be performed at home using the video capsules, as well as other video or web-based training programs. As sanitary measures and confinement regulations could change during the course of the study, participants could also engage in outdoor activities (e.g., walking or cycling). For each exercise session, participants have to report its duration, intensity, and the nature of the activity via a follow-up agenda that will be provided by their training coach. Participants will assess the intensity of their exercise using the modified Borg Rating of Perceived Exertion (RPE) graduated from 0 to 10, where 0 indicates low and 10 high intensity.

#### Physical exercise and cognitive training

The combined multidomain intervention will include home-based physical exercise and cognitive training. Home-based physical exercise will be exactly similar to the conditions described in the previous section. Due to the important variations observed in the short-term effect of exercise on cognitive performances [31], home-based cognitive training will either take place prior to the exercise session or at least 3 h post-exercise training. Participants will be asked to complete their cognitive training in a quiet and comfortable setting without distraction. Both validated computerized cognitive tasks and video capsules will be provided to the participants. Participants will be encouraged to perform at least three sessions per week (30 min/session): two sessions of computerized cognitive tasks and the other one dedicated to the video capsules.

For the computerized cognitive training sessions, the following three tasks will be available: (1) dual-task training that requires participants to maintain and prepare for many response alternatives and to share attention between two concurrent tasks (i.e., Dual-task [32]), (2) inhibition training, which requires the participants to refrain from giving an automatic response (i.e., Stroop task [33]), and (3) working memory training, which asks participants to maintain and update information in working memory to recall items presented earlier (i.e., N-Back task [34]). Each cognitive task lasts approximately 15 min possesses its respective sets of visual stimuli (e.g., letters, numbers, symbols) and matching button symbols. Participants will be instructed to perform the tasks as fast as possible while maintaining accuracy. The three tasks will be presented in a fixed order for all the participants. The level of difficulty of each cognitive task will be gradually increased over the course of the participant’s cognitive training to avoid cognitive task automatization and to maintain stimulation [35]. These computerized tasks will also include visual feedback as well as a histogram of daily performances to encourage improvement and maintain motivation.

The remaining weekly session consists of a strategy-based training performed once a week via video capsules. This training is an adapted version of the MEMO+ program [36]. Participants will be instructed on different mnemotechnics (e.g., face-name association, visual imaging), as well as on age-related changes in memory. In addition, the strategy-based training also includes capsules aiming to help participants develop strategies to manage multiple aspects of their daily life such as sleep, anxiety, and nutrition. Participants will be asked to complete a follow-up agenda and mark days and times when they took part in the various cognitive training sessions to track their cognitive training adherence. Training adherence for computerized tasks will also be monitored online.

#### Remote monitoring and counseling

Once a week, training coaches will call participants over the phone to (1) motivate them to stay engaged in their training and to (2) collect exercise and cognitive training data reported by participants in their follow-up agenda during the previous week. The training coach will also provide and update information about training resources available online, track adherence, and advise participants according to their training characteristics and study training guidelines. Information about physical exercise and cognitive training sessions collected during these calls will allow to track the weekly dose of exercise and cognitive training and to adapt the recommendations given to the participant accordingly.

### Measures

Tests and self-reported questionnaires performed during the preliminary call and the four testing sessions are listed in Table 1. Information about demographics, chronic diseases, comorbidities, and chronic medications will be recorded using a medical questionnaire at baseline. A COVID-19 questionnaire (i.e., QCOVID) will address the social and financial consequences of the COVID-19 pandemic on participants, as well as its impact on their physical activity routines. The QCOVID will also document if the participant and his close circle were tested and/or affected by COVID-19 and ask if they previously developed COVID-19 symptoms, if applicable.

#### Primary outcome—Cognition

Cognitive functioning will be assessed via videoconference, using validated remote versions of paper-pencil neuropsychological assessments [37] and computerized tasks, both performed under the supervision of a research team member. G*eneral cognitive functioning* will be tested using a remote version of the Montreal Cognitive Assessment (MoCA) [38]. The remote version of the MoCA is identical to the original version, to the exception that one question in the orientation subscale is modified (“Now, tell me the name of this place, and which city it is in” was replaced by one question regarding an important sightseeing place in Canada). In addition, the *three main components of cognition* will be assessed with a comprehensive neuropsychological assessment, including both paper-pencil tests and computerized tasks, from which composite z scores will be calculated: executive functions, processing speed, and episodic memory. These different composite *Z*-scores will first be computed by grouping tests and subscores that conceptually assess the same cognitive functions (mean of *z*-scores of different tests and scores). In a second step, internal consistency among each composite *z*-score will be verified with Cronbach’s alphas as done previously (*α* > .7) [39]. The neuropsychological assessment will include the following paper-pencil tests that will be administered in a fixed order: Rey auditory verbal learning test [40], Digit Span, an oral Trail Making Test with three conditions (1—counting from 1 to 20 (maximum of 30s); 2—naming letters from A to Z (maximum of 30 s); 3—alternating between naming numbers and letters in chronological and alphabetical order (e.g., 1-A-2-B) (maximum of 30 s) [41]), Phonological and Semantic Fluency from the D-KEFS battery (Letters P, T, L (60 s maximum); Categories: animals, men’s names (60 s maximum); Switching between naming fruit and furniture (60 s maximum) [42]), and Similarities subtest from the WAIS-IV [43]. These tests are validated and normalized for an older adult population [40, 44, 45]. The Similarities subtest will only be administered at baseline to obtain a measure of crystallized intelligence; it will be optional and performed according to the level of the participant’s fatigue. Participants will also complete a computerized assessment on their tablet or computer [32]. The computerized tasks include a dual-task [32], Stroop task [33], and N-back [34], all in trained and transfer versions (same tasks but with different stimuli) presented in a counterbalanced order between the participants via a Latin square procedure. Subcomponents of each task allow the dissociation of attentional control mechanisms from mere cognitive speed. Importantly, the tasks record response time in milliseconds reducing the likelihood of ceiling effects. These tasks also tend to be more sensitive to training effects. The trained and transfer task comparison allows quantifying participants' ability to transfer cognitive training effects to another task. The computer equipment (i.e., computer or tablet) used by the participant for the computerized tasks will be systematically documented. Participants will be requested to use the same computer equipment for the four testing sessions.

#### Secondary outcome—Physical functions

Physical functions, including *mobility, balance, lower limb muscle strength*, and *estimated cardiorespiratory fitness*, will be assessed using tests and self-reported questionnaires validated for the older adult population. These tests will be performed online and at home under remote supervision. First, mobility will be assessed using the 4-meter walk test and the Timed Up and Go (TUG) performed at spontaneous and fast speeds. During the 4-meter walking test, participants' walking speed will be measured in a straight line over 4 meters. During the TUG, participants will get up from a chair, walk 3 meters, turn around, and sit back in the chair while they are timed. Balance and lower limb muscle strength will be measured by a one-leg balance test (i.e., stay on one leg as long as possible with a maximum duration of 60 s) and a Five-Time-Sit-to-Stand test (i.e., five consecutive sit-to-stand movements done as fast as possible without using arms as leverage), respectively. These mobility and functional tests are all commonly used in a clinical setting and have demonstrated their associations with global cognitive functioning and specific cognitive domains such as processing speed and executive functions [46]. In order to prevent any possibility of falls and secure the participants, before the assessment, each participant will be interviewed about his history of musculoskeletal and sensory disorders (e.g., severe low back pains, recent sprain, vestibular disorders). Also, participants will receive by email a description of each test and its set-up, associated with demonstration videos. Then, the testing set-up will be revised with the participant at the beginning of the functional assessment session to ensure that the participant has enough space and measures the walking tracks for both 4-meter and TUG tests with sufficient precision. Finally, during the testing session, the research team member will verify that they always have suitable visual feedback to ensure the testings' security and reliability. All these dispositions converge with the recently published recommendations for the remote assessment of gait and balance [47]. Finally, the Matthews questionnaire will be used to predict participants’ cardiorespiratory fitness (i.e., VO_2max_) based on sex, age, anthropometric data, and self-reported physical activity level [48].

#### Exploratory outcomes—Mood, anxiety, and health-related quality of life

Mood, anxiety, and health-related quality of life will be assessed using validated self-reported questionnaires provided through online forms. *Mood and anxiety* will be assessed using the 30-item Geriatric Depression Scale [49], the State Anxiety Inventory [50], the Perceived Stress Scale [51], the Perseverative Thinking Questionnaire [52], and Connor-Davidson Resilience Scale 10 [53]. *Health-related quality of life* will be measured using the 12-item Short Form Survey [54].

#### Other variables

Additional variables that might play a potential moderating role on trial outcome include *social activity and support* using the Social and Community Involvement Questionnaire [55] and the Lubben Social Network Scale [56], *sleep* using the Pittsburg Sleep Quality Index [57], and the Berlin Questionnaire [58], *diet* using the Short Diet Questionnaire [59], *cognitive reserve* using years of education and a modified version of the Rami and colleagues’ cognitive reserve questionnaire [60] adapted for French and English by the CIMA-Q team [61], *prior physical activity* using the Physical Activity Scale for the Elderly [62], *anxiety-traits* using Anxiety Sensitivity Index [63], Intolerance to Uncertainty Scale [64], Perceived Vulnerability to Disease [65] and Trait Anxiety Inventory [50], and *gender* using the Bem Sex-Role Inventory [66]. Additionally, the level of *participants’ satisfaction* regarding their evaluations and training will be assessed by a dedicated questionnaire after the 6-month evaluations. These scales will be used as covariables to take into account aspects related to motivation. All self-reported scales and questionnaires will be performed using online forms as described in Table 1.

### Randomization and blinding

All participants who provide their consent, fulfill the inclusion criteria, and complete the baseline assessment will be randomized. Randomization will be performed by a naive staff member who is not involved in any other aspect of the COVEPIC study, upon request of the recruitment coordinator. The staff member will assign the participant to one of the two interventions according to simple randomization with a 1:1 allocation ratio. The Montreal Health Innovations Coordinating Center (MHICC), a research organization independent from the present study team, will generate the randomization sequence (i.e., computer-generated random numbers) that will be protected in a locked sheet. Participants’ intervention group will then be shared to the research staff in charge of training. No modification of group allocation will be allowed. This clinical trial is a single-blinded study. Research personnel performing the outcome assessments at baseline, three, six, and twelve months will be blinded to group allocation. Participants will be aware of the type of training they receive.

### Data analysis

#### Sample size calculation

The Montreal Health Innovations Coordinating Center**’**s biostatistics team completed the sample size calculation. The calculation was based on values available in the literature for physical exercise training alone [67], on different plausible values for the combined physical and cognitive training (since there are no values in the literature for this group), and on clinically relevant effect sizes to detect the cognitive outcome. A sample size of 49 subjects in each group will have 80% power to detect a difference in means of **−** 0.186 (the difference between a physical training alone mean of 0.239 and a combined training mean of 0.425, for an effect size of 0.624), assuming that the common standard deviation is 0.298 using a two-group *t* test with a 0.050 two-sided significance level. Considering a 20% attrition rate observed in our previous exercise studies, 61 participants will be recruited per intervention arm to ensure the targeted 49 participants per group (61 * .80 = 49) for a total of 122 participants.

#### Planned data analysis

The variables in the study will be presented using descriptive statistics. The mean, standard deviation, median, minimum, Q1, Q3, and maximum will be presented for continuous variables. The number and percentage will be presented for nominal/ordinal variables. The statistical tests' assumptions will be examined, and data transformation or non-parametric analyses may be used as appropriate. SPSS and SAS version 9.4 or higher software will be used to conduct the analyses. Statistical tests will be two-tailed, and a *p* value of less than 0.05 will be considered as the indication of statistical significance.

Mean changes in cognitive performance from baseline will be analyzed using a repeated measures analysis of covariance (ANCOVA) model, adjusted for age, sex, education, and the dose of physical exercise, including the effects of the intervention (physical exercise alone or combined with cognitive training) and time (pre at baseline, mid at 3 months, post-intervention at 6 months). Observation of a statistically significant difference in the primary outcome between pre- and post-intervention timepoints will be considered as the evidence of the intervention's efficacy (i.e., primary hypothesis). The interaction between intervention arms (i.e., physical exercise alone or combined with cognitive training) and performance changes from pre to post-intervention will address the added value of the cognitive training compared to physical exercise alone (i.e., secondary hypothesis). Observation of a statistically significant *intervention × time* interaction in the primary outcome will be considered as the evidence that the differential effect operates by the exercises and combined interventions. Secondary and exploratory outcomes will be analyzed as the primary outcome.

##### Dose-Response exploration

The type of activity, its frequency, duration, and intensity (i.e., according to the Borg scale) will be considered to compute the dose of physical exercise. The weekly dose of physical exercise will be converted in kcal and in METs using the Compendium of Physical Activities (i.e., coding scheme classifying specific physical activities by their rate of energy expenditure) [68, 69]. The effect of physical exercise dose on cognitive and physical functions will be evaluated according to participants’ mean weekly dose during the 6 months of intervention. Based on the overall study sample distribution, participants will be classified into three groups: higher, medium, and lower doses of physical exercise (i.e., tercile split). How the dose of physical exercise affects the primary and the secondary outcomes will be addressed by a repeated measures ANCOVA model, adjusted for age, sex, and education, including the effects of the intervention (physical exercise alone or combined with cognitive training), time (pre at baseline, mid at 3 months, post-intervention at 6 months) and dose groups (lower, medium or higher doses). An exploration of the dose-response effect for cognitive training will also be performed within the combined physical exercise and cognitive training intervention group. The dose of cognitive training will be considered according to its mean weekly duration. How the dose of cognitive training affects the primary and the secondary outcomes will be addressed by a repeated measures ANCOVA model, adjusted for age, sex, education, and the dose of physical exercise, including the effects of time (pre at baseline, mid at 3 months, post-intervention at 6 months) and dose groups (lower or higher doses of cognitive training; median split).

### Ethical considerations

This study is conducted in compliance with International Conference on Harmonization Good Clinical Practice (ICH-GCP) and all applicable regulatory requirements. It received the approval of the Research Ethics Board (REB) of the Montreal Heart Institute (FWA00003235; research project: MHI 2019-2785). A formal amendment to the COVEPIC protocol will be done for each modification likely to affect participants’ safety or potential benefits, or involving a significant change in the conduct of the study, its objectives, design, population, intervention, or procedure. Such amendments, as well as any administrative change, even minor, will need the approval by the Montreal Heart Institute’s REB before implementation. In the case of a formal amendment, the research team will submit an update to the Trials Editorial board to modify the published record. No audit of the COVEPIC study is pre-planned. However, the Montreal Heart Institute REB could state at any time during the study completion that an audit of the trial is required. If so, the trial audit will be performed by an independent committee, and the research team will be informed of this audit one month before. Each participant manifesting medical concern or health issues during their participation to the study will be referred to the physician collaborators involved in the COVEPIC trial. They will make a decision about their participation and ensure the appropriate medical follow-ups. At the end of their six months of intervention, the research team will encourage participants to maintain their healthy lifestyle habits and pursue their physical activity routine.

### Dissemination policy

The plan to communicate COVEPIC results includes a systematic revision of the publication and abstract propositions, based on COVEPIC outcomes, by an internal committee that will ensure the appropriateness of the scientific proposition and authorship. To protect the integrity of the major objectives of COVEPIC, data breaking the blind will not be presented prior to the release of the main results. This release will be announced by COVEPIC’s sponsor-investigator. Authorship of the publication reporting the main outcome as well as the ancillary studies will be defined in compliance with the recommendations of the International Committee of Medical Journal Editors [70]. The results of the COVEPIC trial will also be released to the participants, the Centre ÉPIC staff, and more broadly to the general medical community. Finally, no later than three years after COVEPIC ends (i.e., completion of the final assessment by the last participant), COVEPIC’s complete and deidentified dataset will be available for sharing purposes from the principal investigator, under reasonable request.

## Discussion

Older adults are at higher risk to suffer from the direct and collateral consequences of the COVID-19 pandemic [1–3, 10]. In this context, allowing older adults to maintain and/or improve their cognitive and physical health may help to prevent a possible secondary crisis due to the long-term alteration of their health condition. Healthy behaviors are key to enable healthy aging, and environmental strategies are crucial to encourage their adoption. Here, the home-based preventive strategies find a particular resonance as the pandemic context adds a challenge for older adults' health as they no longer have access to sport centers or could be afraid to go outdoors. Both exercise and cognitive training separately have demonstrated enhancements of multiple and specific aspects of cognitive and physical functions [13–20, 23–25], and their combination may lead to a synergistic effect that is likely to reinforce their benefits [26]. Therefore, home-based interventions may help promote older adults’ health while helping to counteract the negative collateral impact of the COVID-19 pandemic. However, the effect of a crisis-adapted intervention needs to be further investigated.

This trial is a research proposition specifically tailored for the COVID-19 pandemic that proposes crisis-adapted lifestyle interventions and remote assessments. More precisely, to further ensure the continuity and the regularity of the exercise training, the proposed trial offers an online video training program, providing a wide range of exercises to help participants maintain their exercise routine even during the lockdown. Also, depending on the pandemic context and their preferences, participants will be encouraged to integrate both outdoor and home-based physical exercise in their routine, as outdoor activities may convey additional benefits [71] and are known to play a critical role in maintaining mobility in old age [72]. Remote interventions have previously demonstrated their efficiency to promote physical activity and cardiorespiratory fitness [21]. In this particular context, the monitoring by a training coach could also allow to combine the moving constraints related to the pandemic with participants’ expectations and possibilities, and therefore support the adaptation of their physical exercise routine. Furthermore, the weekly monitoring could also improve the impact of home-based cognitive training on cognitive functions. Indeed, although cognitive training approaches demonstrated their efficiency to improve specific cognitive functions, mixed results are observed for their home-based versions [73]. Several specificities differentiate the present study cognitive training from a purely home-based training, including the weekly remote monitoring, but also the use of systematic procedures to introduce the concept of cognitive training to the participants and a technology tutorial to ensure their familiarity with the dedicated online tools (i.e., technological tutorial) before the intervention. Together, these measures could help address the issues commonly faced with home-based cognitive training and support adherence, treatment fidelity, and participant compliance while maintaining their motivation.

This randomized trial is the first to assess whether a 6-month home-based preventive intervention, including remote monitoring of physical exercise alone and combined with cognitive training, can promote cognitive and physical functions of community-dwelling older adults during this pandemic period. Additionally, this trial will address the potential added benefits of home-based cognitive training to improve the beneficial impact of regular physical exercise on health outcomes. Finally, the trial will also explore the dose-response relationship in terms of dose of home-based exercise for optimal cognitive and physical benefits.

## Study limitation and development perspectives

Despite its strengths mentioned above, the proposed design presents some limitations. Due to this exceptional pandemic context, some ethical considerations refrained the research team from implementing a control group. Nevertheless, to address this limitation, at least partially, all participants will be included in the final analyses, without exclusion based on participation, to document the dose-response relationship between physical exercise and outcome effects. Additionally, sex was not taken into account in recruitment and randomization processes, although sex differences have been reported on cognition [74], as well as in exercise-training effects on cognition in healthy older adults [75]. Consequently, sex will be considered as a covariate in all statistical analyses. Also, a recent study from Peterman et al. [76] highlights that longitudinal changes in nonexercise prediction equations of cardiorespiratory fitness were associated with changes in cardiorespiratory fitness as measured by standardized VO2_max_ protocols but present significant errors in their predictions. Therefore, the use of nonexercise prediction equations could lead to a significant error in the estimated change of participants' cardiorespiratory fitness.

Future studies using more direct physical fitness assessments and tracking devices for remote monitoring could help better inform on the effect of physical exercise training in seniors. Indeed, adding a direct measure of physical fitness could help inform on participants' physical condition when they engage themselves in the intervention, as well as their progression. Its combination with a measurement of frequency, duration, and intensity of physical exercise sessions provided by tracking devices such as smartwatches or smart garments may shed light on the relationship between exercise training characteristics and the changes in physical fitness over the course of the intervention. This kind of information may substantially improve remote monitoring, helping the training coaches provide resources and recommendations adapted to each participant according to their physical fitness and progression.

Nevertheless, the remote monitoring proposed in COVEPIC, based on a weekly phone call, is an easy method to implement that requires limited resources and represents a promising strategy for the promotion of physical activity regardless of location, access to a sports center or professional supervision. In their report, the WHO alerts the urgent need to invest and give priority to services aiming to promote physical activity in both health and other sectors [11]. In that way, the COVEPIC results, addressing the impact of the remote monitoring on physical activity promotion and on health outcomes, may help to document the benefits of a method as simple as a weekly counseling phone-call. In addition, investigating the added value of cognitive training may also help to document the extent to which the combination of these two interventions (i.e., physical exercise and cognitive stimulation) could lead to greater improvements of cognitive functioning and mobility, and therefore could represent an interesting combination to further improve these crucial functions in aging populations.

## Conclusion

The present study will document the remote monitoring of home-based physical exercise alone and combined physical and cognitive home-based training to promote older adults’ cognitive and physical health during this COVID-19 pandemic period. Home-based interventions remotely monitored represent a promising strategy to promote healthy habits and may be a good alternative to center-based interventions, especially during this COVID-19 pandemic crisis. The results of this trial could open the way for the future development of preventive strategies for isolated and rural geriatric populations who do not typically have access to such interventions and, more broadly, to sport centers.

## Trial status

The COVEPIC study is the fourth version of the protocol validated by the Montreal Heart Institute’s Research Ethics Board in October 2020. The recruitment for the COVEPIC study began in May 2020 and should be completed in August 2021.

## Data Availability

After the study completion, the final dataset will be available for investigators. It will also be available for the public from the corresponding author on reasonable request.

## Abbreviations

ANCOVA: Analysis of covariance
CIMA-Q: Consortium pour l'identification précoce de la maladie d'Alzheimer
COPD: Chronic obstructive pulmonary disease
COVID-19: Coronavirus disease 2019
D-KEFS: Delis-Kaplan Executive Function System
ICH-GCP: Conference on Harmonization Good Clinical Practice
METs: Metabolic Equivalent of Task
MMSE: Mini-Mental State Examination
MoCA: Montreal Cognitive Assessment
PARQ+: Physical Activity Readiness Questionnaire-Plus
Q1: First quartile
Q3: Third quartile
QCOVID: COVID-19 questionnaire
RPE: Rating of Perceived Exertion
TUG: Timed Up and Go
WAIS-IV: Wechsler Adult Intelligence Scale Fourth Edition
